# Development of Sustained Release Baricitinib Loaded Lipid-Polymer Hybrid Nanoparticles with Improved Oral Bioavailability

**DOI:** 10.3390/molecules27010168

**Published:** 2021-12-28

**Authors:** Md. Khalid Anwer, Essam A. Ali, Muzaffar Iqbal, Mohammed Muqtader Ahmed, Mohammed F. Aldawsari, Ahmed Al Saqr, Mohd Nazam Ansari, M. Ali Aboudzadeh

**Affiliations:** 1 Department of Pharmaceutics, College of Pharmacy, Prince Sattam Bin Abdulaziz University, Al-Kharj 11942, Saudi Arabia; mo.ahmed@psau.edu.sa (M.M.A.); moh.aldawsari@psau.edu.sa (M.F.A.); a.alsaqr@psau.edu.sa (A.A.S.); 2 Department of Pharmaceutical Chemistry, College of Pharmacy, King Saud University, Riyadh 11451, Saudi Arabia; esali@ksu.edu.sa (E.A.A.); muziqbal@ksu.edu.sa (M.I.); 3 Bioavailability Laboratory, College of Pharmacy, King Saud University, Riyadh 11451, Saudi Arabia; 4 Department of Pharmacology and Toxicology, College of Pharmacy, Prince Sattam Bin Abdulaziz University, Al-Kharj 11942, Saudi Arabia; m.ansari@psau.edu.sa; 5 CNRS, Institut des Sciences Analytiques et de Physico-Chimie pour l’Environnement et les Matériaux, University Pau & Pays Adour, E2S UPPA, IPREM, UMR5254, 64000 Pau, France

**Keywords:** baricitinib, bioavailability, encapsulation, hybrid nanoparticles, poly(d,l-lactide-coglycolide), stearin

## Abstract

Baricitinib (BTB) is an orally administered Janus kinase inhibitor, therapeutically used for the treatment of rheumatoid arthritis. Recently it has also been approved for the treatment of COVID-19 infection. In this study, four different BTB-loaded lipids (stearin)-polymer (Poly(d,l-lactide-co-glycolide)) hybrid nanoparticles (B-PLN1 to B-PLN4) were prepared by the single-step nanoprecipitation method. Next, they were characterised in terms of physicochemical properties such as particle size, zeta potential (ζP), polydispersity index (PDI), entrapment efficiency (EE) and drug loading (DL). Based on preliminary evaluation, the B-PLN4 was regarded as the optimised formulation with particle size (272 ± 7.6 nm), PDI (0.225), ζP (−36.5 ± 3.1 mV), %EE (71.6 ± 1.5%) and %DL (2.87 ± 0.42%). This formulation (B-PLN4) was further assessed concerning morphology, in vitro release, and in vivo pharmacokinetic studies in rats. The in vitro release profile exhibited a sustained release pattern well-fitted by the Korsmeyer–Peppas kinetic model (R^2^ = 0.879). The in vivo pharmacokinetic data showed an enhancement (2.92 times more) in bioavailability in comparison to the normal suspension of pure BTB. These data concluded that the formulated lipid-polymer hybrid nanoparticles could be a promising drug delivery option to enhance the bioavailability of BTB. Overall, this study provides a scientific basis for future studies on the entrapment efficiency of lipid-polymer hybrid systems as promising carriers for overcoming pharmacokinetic limitations.

## 1. Introduction

Baricitinib (BTB) is a small molecule that inhibits Janus-associated kinase (JAK) and therapeutically is used for a group of severe inflammatory disorders including resistant rheumatoid arthritis (RA), systemic lupus erythematosus, auto-inflammatory disease, dermatologic disorders, graft versus host disease and uncontrolled infections [1,2]. Recently, it has been reported that BTB interrupts the signalling of multiple cytokines implicated in coronavirus disease-19 (COVID-19) immunopathology. It may also have antiviral efficacies by targeting host factors that viruses rely on for cell entry and by restraining type I interferon driven angiotensin-converting-enzyme-2 up-regulation [3]. Therefore, it has also obtained Emergency Use Authorization (EUA) for the treatment of suspected or laboratory-confirmed critically ill COVID-19 patients solely or together with remdesivir (RDV). BTB is potent and highly effective against JAK 1 and JAK 2 enzymes with half-maximal inhibitory concentration (IC_50_) values of 5.9 nM and 5.7 nM, respectively, and barely effective counter to JAK 3 (IC_50_ = 400 nM) [4]. Being a small molecule (molecular weight: 371.42 Da), its intra-cellular penetration is appropriate, thus, it can be orally delivered which significantly facilitates regular administration. The oral absorption of BTB is fast with peak plasma concentration achieved within 1 h, but the bioavailability of orally administered BTB varies between different species and were from 48% (dogs), 54% (rats), 47–68% (monkey) to 79% in human (EMEA Assessment report). Its volume of distribution is 76 L with plasma and serum protein binding about 50% and 45 %, respectively and a half-life of nearly 12 h. 

As mentioned in the assessment report submitted for regulatory approval, BTB belongs to the biopharmaceutical classification system (BCS) class III substance, which means that it is a highly soluble and poorly permeable drug [5]. In addition, as per the DrugBank identification report, BTB has both solubility (0.357 mg/mL in water) and permeability (Log P = 1.08) issues [6] and drugs with these characteristics may exhibit low (and inconsistent) bioavailability affecting the efficiency of therapeutic benefits [7].

One approach to enhance the bioavailability of bioactive compounds is the encapsulation process in a way that using various coating materials enables target delivery and controlled release [8,9]. Choosing the suitable coating material is vital in the encapsulation process, owing to its impact on target delivery and controlled release, and consequently, on the bioaccessibility of active components. Among different choices, hybrid materials based on polymers are promising therapeutics systems. These hybrid materials have already demonstrated excellent commitment in addressing and offering solutions to the existing challenges in priority areas such as human health, environment and energy [10,11,12]. However, their design, performance, and practical applications are still ambitious [13,14].

Lipid-polymer hybrid nanoparticles (LP-NPs) are novel hybrid materials that have gained a lot of attention in recent years and they have been developed to achieve an improved therapeutic effect with the least adverse effect [15,16]. Due to their particular core–shell structure, LP-NPs exhibit good storage stability, controlled release profiles due to the polymer core, enhanced therapeutic potency, and biocompatibility because of the lipid–PEG and lipid layers [17]. LP-NPs are effective in encapsulating the hydrophobic molecules with a higher drug payload than biopolymer-based nanoparticles due to their nano-range size and large surface areas [18]. In literature, chitosan polymer-based LP-NPs have been reported to improve drug stability and to improve the oral bioavailability of poorly water-soluble drugs [19,20,21].

Due to the biodegradable and biocompatible nature of Poly(d,l-lactide-coglycolide) (PLGA), it is considered a smart polymer and is being used extensively for the enhancement of solubility and bioavailability of poorly soluble drugs [22,23,24,25]. In addition, PLGA is non-toxic and non-immunogenic and approved by US FDA for pharmaceutical and biomedical applications [26]. However, there are limited reports of PLGA-based LP-NPs for improving the solubility and permeability of bioactive compounds. Recently, PLGA-based LP-NPs has been successfully used for the bioavailability enhancement of poorly water-soluble drugs e.g., paclitaxel (PLGA as polymer and stearyl amine, soya lecithin as lipids) and hydroxycamptothecin (PLGA as polymer and 1,2-distearoyl-*sn*-glycero-3-phosphoethanolamine-*N*-(methoxy(polyethylene glycol)-2000) (DSPE-PEG_2000_), and lecithin as lipid) [27,28] which resulted for improvement of intracellular uptake of drugs to overcome the multidrug resistance in cancer and enhancement of antitumour activity [29,30]. Previously, PLGA-based BTB nanoparticles have been developed and characterised to show their sustained release performance but without in vivo bioavailability study [31]. To the best of our knowledge, no study has been reported for fabrication of PLGA-based LP-NPs having tristearin and soyalecithin (SL) as lipids aiming at enhancing the bioavailability of poorly soluble and low permeable drugs.

The main goal of this work is to promote the oral bioavailability of BTB through its encapsulation in PLGA-based LP-NPs carriers. Therefore, four different BTB-loaded LP-NPs (B-PLN1 to B-PLN4) were developed by varying the lipid content. Analysing the formulations in terms of physicochemical properties and encapsulation efficiency allowed us to select the optimised nanoparticle carrier which was investigated further in terms of morphology, in vitro release, and in vivo pharmacokinetic studies in rats. The findings in this study may be further sculpted into new encapsulation strategies by employing hybrid materials.

## 2. Results and Discussion

### 2.1. Particles Characterisation

BTB-loaded PLGA-based LP-NPs (B-PLNs) were prepared by the single-step nanoprecipitation method, consisting of PLGA polymer and tristearin lipid as core and shell parts, respectively. Sonication was employed using a probe sonicator, which produces high-intensity ultrasonic waves that break the big particles into nanoparticles. To optimise B-PLNs, the amount of tristearin (lipid) was varied, utilising a fixed amount of PLGA (polymer) and SL (surfactant). The mean size, PDI and ζP of the B-LPNs at different lipid contents (50–200 mg) are shown in Table 1. The particle size of different formulae (B-PLN1 to B-PLN4) was obtained in the range of 205 ± 5.2 to 272 ± 7.6 nm. The lipids covered the PLGA core, which thickened the shell, thereby increasing the particle size. The purpose of the selection of tristearin as lipid in this formulation as it shows better cellular uptake, low toxicity and greater immune response. [32,33]. The PDI values of PLNs were measured in the range of 0.170–0.299, which indicates a homogenous population of PLNs [34,35]. ζP is the key parameter for the evaluation of the stability of colloidal dispersion. The ζP of the prepared PLNs were measured in the range of −21.1 to −36.5 mV, negative values of ζP are due to negatively charged SL [36]. It is believed that the values of ζP ≥ ±30 mV suggest the formation of stable particles, nevertheless, it has to be mentioned that the usual Smoluchowski method to determine ζP is only valid for hard spheres [37]. In this case, due to the soft nature of B-PLNs, ζP calculated by conventional analysis does not reflect the state of agglomeration or stability. Our soft particles were stable despite in some cases (B-PLN1 and B-PLN2) ζP < ±30 mV [38]. As can be seen in Table 1, the particle size, PDI and ζP of formulated B-PLNs increase as the lipid content increases.

### 2.2. Percent Drug Entrapment (%EE) and Loading (%DL)

Entrapment efficiency gives an idea about the amount of drug that is successfully entrapped/adsorbed into nanoparticles. Typically, an excellent drug carrier should have high entrapment efficiency (EE). High EE (above 70%) can increase the efficacy of the drug delivery system and decrease the side effects of the drug [39,40]. The %EE and %DL of B-PLNs (B-PLN1 to B-PLN4) were measured in the range of 45.9 ± 1.9 to 71.6 ± 1.5% and 2.87 ± 0.42 to 6.80 ± 0.81%, respectively (Table 1). The highest drug entrapment (71.6 ± 1.5%) was found in the case of B-PLN4, a large amount of lipid (200 mg, stearin) formulated in this sample is supposed to prevent the diffusion of the drug from the polymeric core, thereby, enhancing the entrapment of drug [41].

### 2.3. DSC Studies

**Figure 1:**
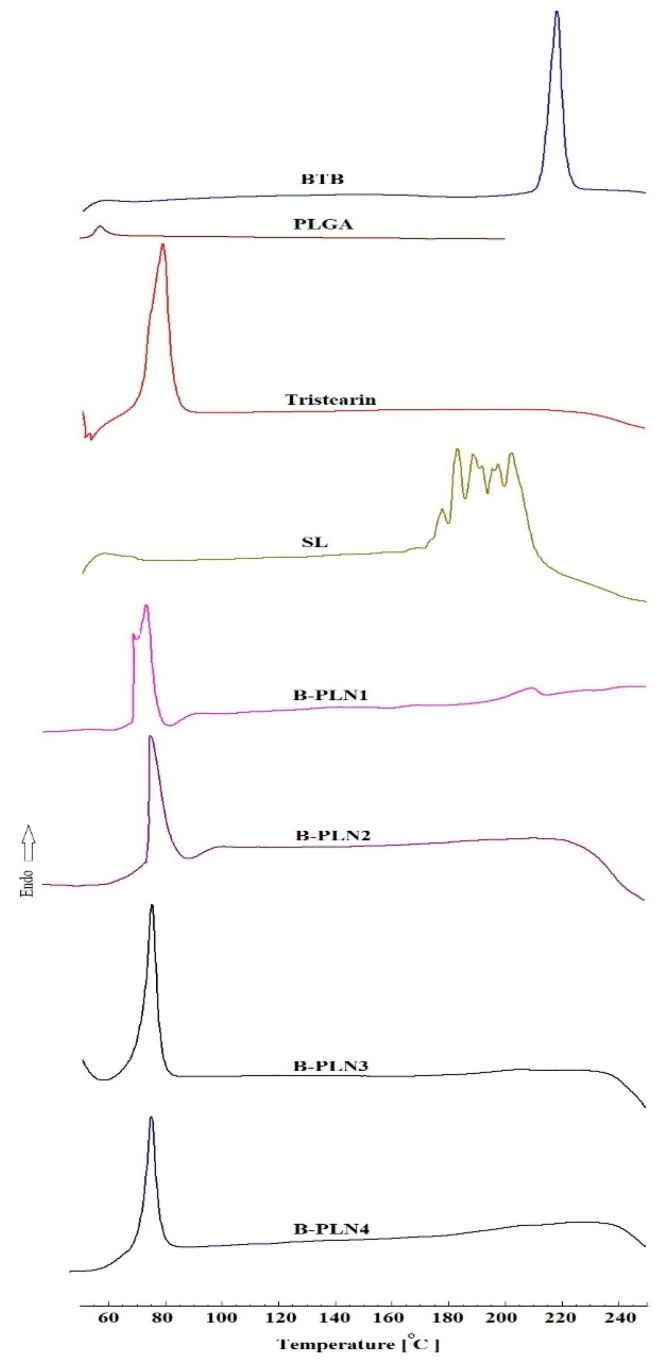
Comparative DSC thermograms of BTB, PLGA, stearin, SL and formulated lipid-polymer nanoparticles (B-PLN1 to B-PLN4).

DSC thermal studies were performed to investigate the compatibility of drug and excipients (PLGA, tristearin, and SL). The DSC spectra of BTB, PLGA, tristearin, SL and their B-PLNs (B-PLN1-B-PLN4) are presented in Figure 1. The DSC spectra of pure BTB drug exhibited a sharp endothermic peak at 217.97 °C, which confirmed the purity and crystallinity of the drug [31]. PLGA showed a glass transition temperature at 58 °C. The DSC spectrum of tristearin exhibited a distinct endothermic peak at 79 °C, whereas SL showed a merge of multiple peaks between temperatures 180 to 205 °C [42,43]. The endothermic peak of pure BTB were absent in all B-PLNs (B-PLN1-B-PLN4), which clearly indicated successful encapsulation of the drug [44]. The peak associated with tristearin could be seen in all formulations, due to the covering of lipid on the polymeric core.

### 2.4. FTIR Studies

**Figure 2:**
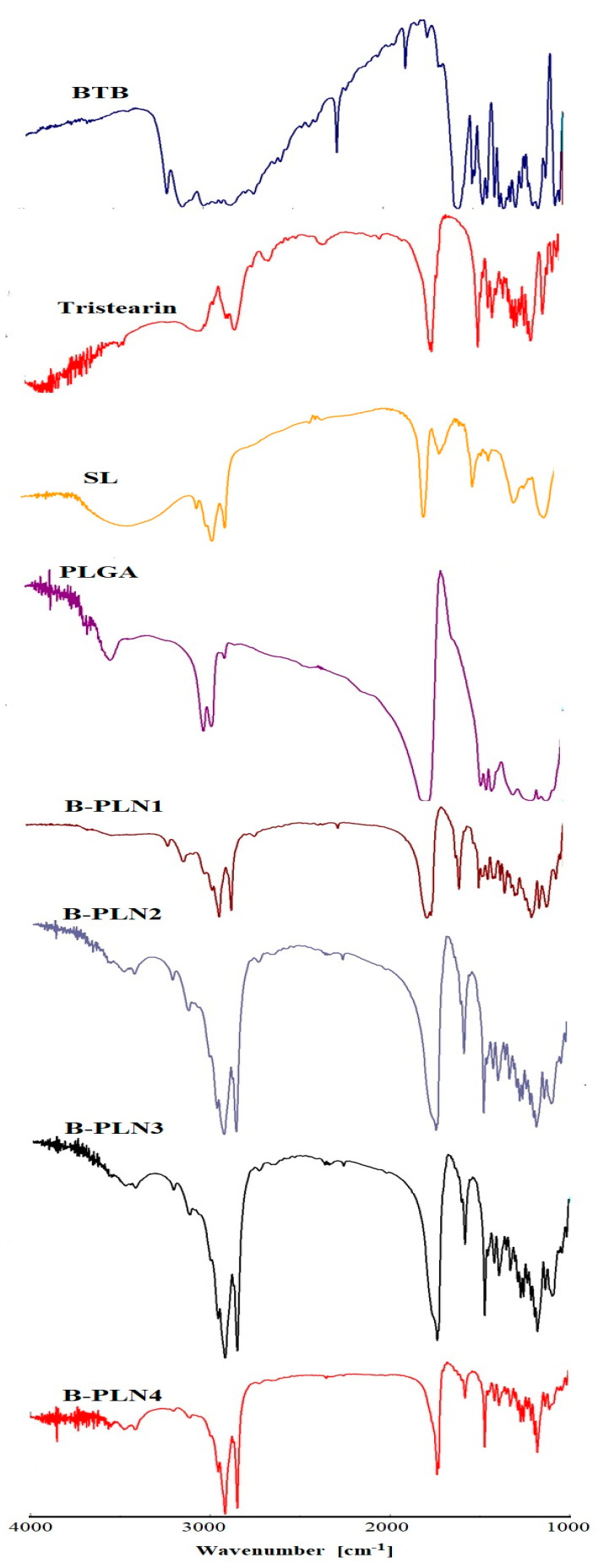
Comparative FTIR spectra of BTB, stearin, PLGA, SL and formulated lipid-polymer nanoparticles (B-PLN1 to B-PLN4).

FTIR spectral studies were performed to investigate the possible chemical interactions between drug and excipients (PLGA, tristearin and SL). The FTIR spectra of BTB, PLGA, tristearin, SL and their corresponding B-PLNs (B-PLN1 to B-PLN4) are presented in Figure 2. The FTIR spectra of pure BTB assigned various characteristics peaks at wave numbers 3207 cm^−1^ (N-H stretching), 3119 cm^−1^ (aromatic =C-H stretching), 2842 cm^−1^ (-C-H stretching), 2263 cm^−1^ (-C=N stretching). FTIR spectra of SL showed a characteristic peak at 2924 cm^−1^ and 2856 cm^−1^ (-C-H stretching), 1738 cm^−1^ (-C=O stretching). FTIR spectra of stearin showed a characteristic peak at 2922 cm^−1^ and 2852 cm^−1^ (-C-H stretching), 1729 cm^−1^ (-C=O stretching) [45]. The FTIR spectra of PLGA indicated a strong peak at 1753 cm^−1^ (C=O stretching). The characteristic peaks of stearin and SL were observed in the spectrum of B-PLNs (B-PLN1 to B-PLN4), suggesting that BTB was successfully loaded inside the lipid shell.

### 2.5. XRD Studies

**Figure 3:**
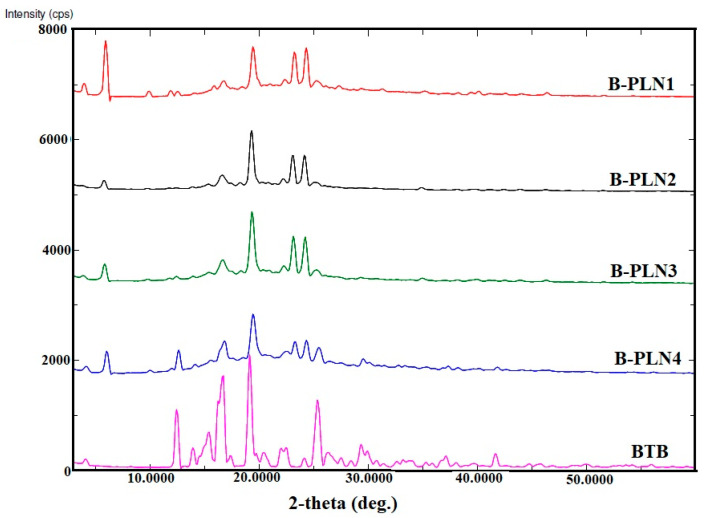
XRD spectra of BTB and formulated lipid-polymer nanoparticles (B-PLN1-B-PLN4).

XRD is a frequently used technique for the characterisation that provides the information regarding crystalline and amorphous nature of nanoparticles. Comparative XRD spectra of pure BTB and their polymer-lipid hybrid nanoparticles (B-PLN1 to B-PLN4) are shown in Figure 3. The XRD spectra of pure BTB shows various intense peaks at 12.5° (2θ), 13.6° (2θ), 15.2° (2θ), 17.2° (2θ), 18.9° (2θ) and 26.6° (2θ), which revealed its crystalline nature [31]. The intense XRD peaks of pure BTB were reduced in intensity, broadened or diffused in all B-PLN samples (B-PLN1 to B-PLN4), which clearly indicated amorphisation of the drug, probably due to encapsulation inside polymer and lipid matrix. The polymeric encapsulation layers hinder the drug, which is then not able to crystallise at the solid–air interface. As the result, the coating layer introduces another solid–solid boundary. This process is called amorphous solid dispersion and is certainly the consequence of disrupting intermolecular interactions in the drug's crystal lattice and assembling drug–polymer interactions [46]. Some extra peaks associated with excipients can be seen in all formulations.

### 2.6. In Vitro Release Studies

**Figure 4:**
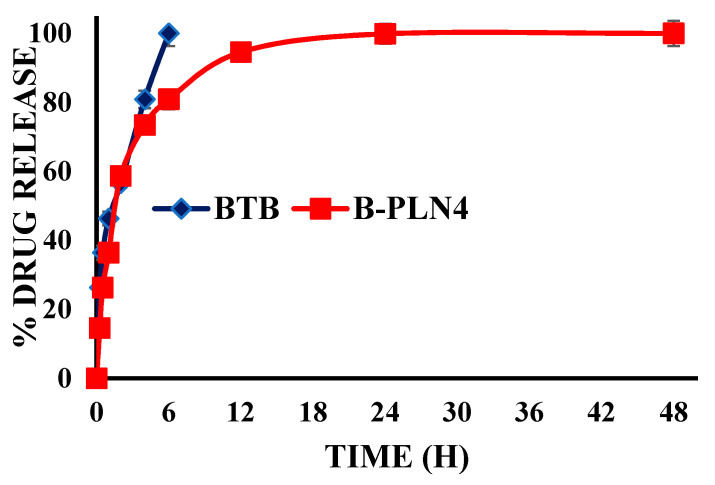
Comparative in vitro release profile of pure drug BTB and optimised B-PLN4 formulation.

Comparative in vitro release profiles of pure BTB and B-PLN4 are shown in Figure 4. The first phase burst release of BTB was observed in B-PLN4 formulation in the first 4 h, probably due to surface adsorbed drug on nanoparticles. Thereafter, the second phase has shown sustained release of BTB from lipid-coated polymer hybrid nanoparticles till 48 h. Sustained release of drug was observed due to slow release of drug by diffusion from tristearin and PLGA matrix. However, 100% drug released was observed from pure BTB in the first six hours of the study. The sustained release of BTB may help reduce the frequency of oral administration and in chronic arthritis treatment. The drug release data of optimised formulation (B-PLN4) was treated by employing various kinetic models viz; zero-order (R^2^ = 0.4848), first-order (R^2^ = 0.6525), Higuchi model (R^2^ = 0.7546) and Korsmeyer–Peppas model (R^2^ = 0.879). The correlation of coefficient of various models indicated that formulation B-PLN4 was best fitted with the Korsmeyer–Peppas model (R^2^ = 0.879) comparison to other kinetic models. Korsmeyer–Peppas model indicated a release simultaneously by diffusion of water into the matrix, swelling of matrix and dissolution of matrix. Additionally, the release data were fitted to the Korsmeyer–Peppas model at a dose of 100% drug release showed the release exponent *n* = 0.3619. The n value less than 0.43 in the spherical encapsulation shape indicates a Fickian diffusion release of the drug [47,48,49,50]. The initial burst release followed by sustained release of drug during dissolution also supports the goodness of Korsmeyer–Peppas kinetic models [51].

### 2.7. Morphology

**Figure 5:**
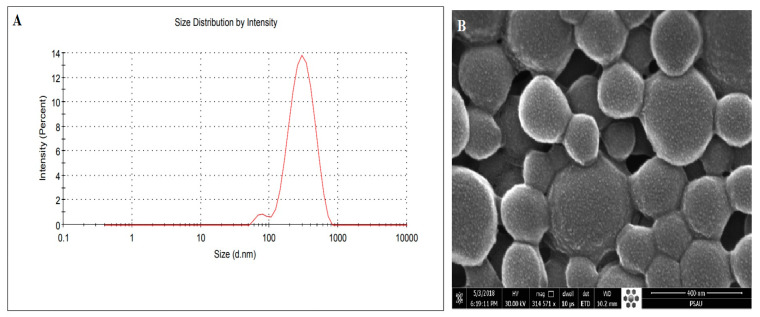
Size distribution plot derived by DLS method (**A**) and SEM images (**B**) of optimised lipid-polymer nanoparticle system (B-PLN4).

SEM images of optimised formulae B-PLN4 represents spherical shapes and with a rough surface. Rough surfaced and agglomerated particles could be seen, probably due to the melting of lipid matrix as stearin melts around 72 °C [52] (Figure 5). The size of the particles was approximately identical as measured by the DLS technique.

### 2.8. In Vivo Pharmacokinetic Study

**Figure 6:**
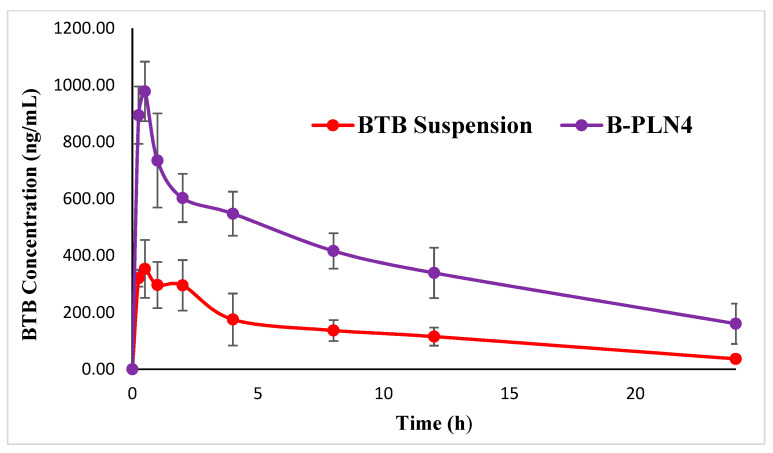
Comparative plasma concentration versus time profile of BTB after oral administration of BTB pure suspension and BPLN4 (1 mg/kg) in rats (*n* = 6).

**Figure 7:**
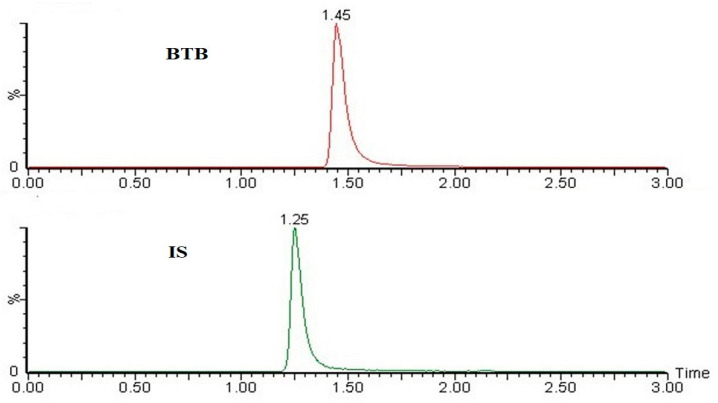
Representative MRM chromatogram of BTB and IS at T_max_ after oral administration of 1 mg/kg of B-PLN4 formulation in rats.

The pharmacokinetic parameters calculated after oral administration of pure BTB suspension and B-PLN4 formulation (described by a non-compartmental pharmacokinetic analysis) are presented in Table 2. At the administered dose, both C_max_ (*p* < 0.001) and AUC_0–24_ (*p* < 0.005), AUC_0–∞_ (*p* < 0.05) values were significantly higher in B-PLN4 formulation compared to pure BTB suspension, without any change in the T_max_ value. This enabled high circulation capability of B-PLN4 formulation and therefore the resulted relative bioavailability was 3-fold higher than pure BTB suspension. Similar results have been also observed in previous PLGA-based polymer-lipid hybrid nanoparticles [27,28]. Moreover, the half-life of B-PLN4 formulation (11.7 h) was higher (although it was not significant) than the plane BTB suspension (8.2 h), indicating that B-PLN4 formulation not only increases the bioavailability of BTB but also facilitates long-term retention i.e., sustain release performance. This was also confirmed by comparing the values of AUC_0–∞_/AUC_0–24_ for B-PLN4 (only 75 %) to the one of pure suspension (87 %), implying that a substantial concentration of BTB was still present at the last time point (24 h) and additional timepoint up to 48 h was necessary to be covered for sampling to calculate accurate pharmacokinetic profiles, which is also evident in plasma concentration-time profile (Figure 6). BTB appearance in the circulatory system after administration of B-PLN4 was composed of two steps: the release of the drug from B-PLN4 and the absorption of BTB into the central compartment. The B-PLN4 formulation underwent a slower distribution rate as compared to BTB pure suspension as evidenced from the prolonged half-life. These results confirm that the release of BTB from B-PLN4 formulation was the rate-determining step owing to the protective effects of polymer-lipid hybrid nanoparticles suggesting that BTB loaded lipid polymer nanoparticles were able to enhance BTB in vivo bioavailability. Moreover, the results of the in-vitro release profile of B-PLN4 formulation were also comparable with our pharmacokinetic results. The representative multiple reaction monitoring (MRM) mode chromatograms of BTB and IS (rivaroxaban) after oral administration of BTB (1 mg/Kg) is presented in Figure 7.

## 3. Materials and Methods

### 3.1. Materials

BTB was purchased from “Mesochem Technology” Beijing, China. Tristearin (Dynasan 188), PLGA and Soyalecithin (SL) were purchased from “Sigma Aldrich, St. Louis, MO, USA”. All other chemicals and solvents were used as received.

### 3.2. Preparation of BTB-Loaded PLGA-Lipid Hybrid Nanoparticles

The PLGA-lipid hybrid nanoparticles were prepared using the single-step nano-precipitation method followed by emulsification [22]. Briefly, PLGA (50 mg) and tristearin (lipid) were dissolved in 10 mL of dichloromethane, further drug BTB was added in the above formed organic phase. Separately, the aqueous phase was prepared by adding SL in 20 mL of distilled water. The prepared organic phase was emulsified by adding in aqueous phase dropwise (0.3 mL/min), and sonicated on probe sonicator “(probe # 423, model CL-18, Fisher scientific; Massachusetts, MA, USA)” for 3 min, on/off cycles 10 secs at 60% W power efficiency. The produced emulsion was left under stirring overnight at room temperature to evaporate organic solvent. The resultant suspension was centrifuged “(HermleLabortechnik, Z216MK, Wehingen, Siemensstraße, Germany)” at high speed (12,000 rpm) to separate non-entrapped drugs. The collected sediment was further washed three times with double distilled water and then lyophilised. Four formulations were developed by varying the tristearin content (50–200 mg) (Table 3).

### 3.3. Particles Characterisation

The particle size, polydispersity index (PDI) and zeta potential (ζP) of synthesised LP-NPs were measured using the DLS method “(Zetasizer Nano ZS instrument, Malvern Instruments, Worcestershire, UK)” at room temperature (25 ± 2 °C). The light scattering angle of measurement was set at 90°. All the measurements were performed in triplicate and data was presented in mean ± SD (*n* = 3).

### 3.4. Percent Drug Entrapment (%EE) and Loading (%DL)

The freshly prepared dispersion was centrifuged “(HermleLabortechnik, Z216MK, Wehingen, Siemensstraße, Germany)” at 12,000 rpm for 15 min to separate the solid sediment. The obtained supernatant was filtered and diluted appropriately, and analysed for free drug content by UV spectroscopy “(Jasco UV spectrophotometer V-630 Japan)”. The %EE and %DL were measured using the following equation:

$$\%EE=\frac{Total\;BTB\;loaded-free\;BTB\;in\;supernatant}{Total\;BTB\;loaded}$$

$$\%DL=\frac{Intially\;BTB\;added-free\;BTB\;in\;supernatant}{Total\;weight\;of\;lipid\;polymer\;NPs}$$

### 3.5. Differential Scanning Calorimetry (DSC) Studies

The thermal properties of pure BTB, PLGA, tristearin, SL and their B-PLNs (B-PLN1-B-PLN4) were examined by DSC “(DSC N-650; Scinco, Seoul, Korea). Accurately weighed (5 mg) of each sample was pressed into a hermetically sealed aluminium pan, placed in a DSC sample holder, and heated for a temperature ranging from 50 °C to 250 °C at a heating rate of 10 °C/min. The instrument was continuously purged with inert nitrogen gas with a flow rate of 20 mL/min during the experiment.

### 3.6. Fourier Transform Infrared (FTIR) Studies

FTIR spectra of pure BTB, PLGA, tristearin, SL and their B-PLNs (B-PLN1-B-PLN4) were recorded using “FTIR spectrometer (Jasco FTIR Spectrophotometer, Tokyo, Japan)”. For the preparation of the sample, each sample was diluted with potassium bromide (KBr) crystal (1:10, *w*/*w*) to prepare pellets. FTIR spectra were recorded in the range of 4000 to 1000 cm^−1^, and peaks were interpreted using “spectral manager” software.

### 3.7. X-ray Diffraction (XRD) Studies

XRD studies of pure BTB and their polymer-lipid hybrid nanoparticles (B-PLN1-B-PLN4) were recorded using “Ultima IV Diffractometer (Rigaku Inc., Tokyo, Japan at College of Pharmacy, King Saud University, Riyadh, KSA)”. The samples equivalent 200 mg of pure BTB were spread on the sample holder and scanned in the range of 0–60° (2θ) at a scan rate of 4°/min.

### 3.8. In Vitro Release Studies

In vitro release studies of pure BTB drug and synthesised polymer-lipid hybrid nanoparticles (B-PLN1-B-PLN4) were performed using dialysis bag “(cut off of 12 kda)” method over a period of 48 h. Based on preliminary evaluation, B-PLN4 formulae was optimised for release and pharmacokinetic studies. Briefly, pure BTB and B-PLN4 (equivalent to10 mg BTB) was dispersed in a dialysis bag containing 10 mL of phosphate buffer (pH 6.8) and shaken on a biological shaker “(LBS-030S-Lab Tech, Kyonggi, Korea)” at 100 rpm and 37 °C [31]. The supernatant of the samples was collected, centrifuged (12,000 rpm) “(HermleLabortechnik, Z216MK, Wehingen, Germany)” and analysed at 224 nm on different time points (0, 0.25, 0.5, 1, 2, 4, 6, 12, 24 and 48 h). Each analysis was performed in triplicate. To check the kinetic release pattern of BTB from B-PLN4, the obtained release data were fitted with various kinetic models viz. zero order, first order, Higuchi’s and Korsemeyer–Peppas kinetic models as follow:

Zero order: (Q_t_ = Q_0_ + K_0_t)

First Order: (ln Q_t_ = ln Q_0_ + K_1_t)

Higuchi: (Q_t_ = K_H_t^1/2^)

Korsmeyer–Peppas: (Q_t_/Q_∞_ = K_K_ t^n^)

### 3.9. Morphology

The morphology of optimised formulae, B-PLN4 was viewed using Scanning Electron Microscopy (SEM) “(JEOL JSM-5900-LV, Tokyo, Japan)”. The sample was homogenously spread and coated with gold-metal in a thin film coater under vacuum “(Quorum Q150R S, East Sussex, UK)”. The pre-treated sample was then bombarded with an electron beam and the interaction resulted in the formation of secondary electrons called auger electrons. From this interaction between the electron beam and the specimen’s atoms, only the electrons scattered at ≥90° were selected and surface topography was taken at 15 kV acceleration voltage and 314,571× magnification [23].

### 3.10. Bio-Analytical Methods

BTB was quantified in rat plasma samples by a slight modification of our previous reported UPLC-MS/MS method [50]. The precursor to production ion transition of 372.07 > 251.14 and 440.04 > 4144.9 (quantifier) were used BTB and internal standard (rivaroxaban d-5) quantification in multiple reactions monitoring mode for detection. The capillary voltage was 3.9 kV and the cone voltage and collision energy were set at 50 V and 30 eV for BTB and 46 V and 28 eV for IS, respectively. Before analysis, the method was validated and all parameters were within the acceptable range mentioned in the guideline for bioanalytical method validation.

### 3.11. Pharmacokinetic Studies

The comparative pharmacokinetic study of newly synthesised B-PLN4 formulation against normal BTB suspension was performed in rats. The experimental protocol involved twelve healthy adult male Wistar albino rats (*n* = 6, weight 200 ± 20 g), randomly divided into two groups: BTB suspension dispersed in 0.5% *w*/*v* carboxy methyl cellulose (1 mg/kg, p.o.) and B-PLN4 formulation (1 mg/kg, p.o.). The animals were received from the “Animal Care Centre, College of Pharmacy, Prince Sattam Bin Abdulaziz University, Alkharj” and were kept under the recommended conditions with access to food and water ad libitum. The experimental protocol was reviewed and approved by “Research Ethics Committee, Prince Sattam Bin Abdulaziz University (Approval number: BERC 003-03-21)” and the study was performed following all applicable international guidelines for animal handling and use. The animals were fasted overnight and the blood samples were collected in pre-heparinised tube was at a fixed time span (0, 0.25, 0.5, 1, 2, 4, 8, 12 and 24 h) after administration of respective formations. All blood samples were centrifuged at 4500× *g* for 5 min to separate the plasma and all the samples were safely stored in a deep freezer (80 ± 10 °C) until analysis by UPLC-MS/MS.

The non-compartmental pharmacokinetic model was selected to calculate the different pharmacokinetic parameters using “WinNonlin software, Pharsight Co., Mountain View, CA, USA”. All the results were presented as mean ± standard deviation (SD). The parameters; peak plasma concentration (C_max_), time to reach peak concentration (T_max_), area under cure (AUC) [(AUC_0–24_) (AUC_0–∞_)], elimination half-life (T_½_) and rate constant (kz), mean residence time (MRT) were calculated. Unpaired *t*-test was used to compare the results between normal BTB suspension and B-PLN4 formulation (*p* < 0.05) was considered statistically significant.

### 3.12. Statistical Analysis

“One-way ANOVA using Dunnett’s test. However, an unpaired *t*-test was used for the statistical evaluation of pharmacokinetic parameters. The GraphPad InStat software was used for statistical analysis, and *p* < 0.05 was considered significant”.

## 4. Conclusions

The BTB-loaded lipid-polymer hybrid NPs were prepared using PLGA, lecithin and stearin through a single-step nano-precipitation method to enhance the bioavailability and sustained release profile of BTB. The PLGA precipitated forming hydrophobic core to encapsulate poorly soluble drug (BTB). The SL and stearin assembled around the PLGA polymer core to form a lipid layer shell. The influence of different concentrations of lipid (tristearin) and soya lecithin (surfactant) on particle sizes, zeta potentials and % EE were assessed. Further, all four B-PLN (B-PLN1 to B-PLN4) formulations were characterised by the DSC, FTIR and X-ray diffraction studies. The optimised B-PLN4 formulation was morphologically characterised by SEM study. In vitro release profile of BTB from lipid-coated polymer hybrid nanoparticles showed slow release of drug by diffusion from tristearin and PLGA matrix. In vivo pharmacokinetic study showed that the lipid-coated polymer hybrid nanoparticles prolonged circulation time when compared with pure BTB suspension which results in a 3-fold increase in bioavailability of B-PLN4 formulation. Overall, it can be concluded that the lipid-coated polymer hybrid NPs can open up a new route for drug delivery with improved potential. However, more extended sampling time points need to be added in future pharmacokinetic studies to cover the better pharmacokinetic profile of BTB formulations.

## Figures and Tables

**Table 1:** Physicochemical characterisation of nanoparticles (B-PLNs).

Sample	Size (nm ± SD)	PDI	ζP (±mV)	%EE	%DL
B-PLN1	205 ± 5.2	0.170	−21.1 ± 2.1	57.8 ± 1.1	6.80 ± 0.81
B-PLN2	231 ± 4.3	0.202	−26.5 ± 2.7	45.9 ± 1.9	6.51 ± 1.02
B-PLN3	259 ± 8.6	0.299	−32.4 ± 1.8	68.6 ± 1.9	5.08 ± 0.95
B-PLN4	272 ± 7.6	0.225	−36.5 ± 3.1	71.6 ± 1.5	2.87 ± 0.42

**Table 2:** Pharmacokinetic Parameters after a single oral dose of pure BTB suspension and B-PLN4 administration (1 mg/kg in rats).

Pharmacokinetic Parameters	Pure BTB Suspension	B-PLN4
	Mean ± SD, (*n* = 6)	Mean ± SD, (*n* = 6)
C_max_ (ng/mL)	404 ± 58	1020 ± 34 ***
T_max_ (h)	0.5	0.5
AUC_0–24_ (ng·h/mL)	3091 ± 720	9030 ± 1487 **
AUC_0–∞_ (ng·h/mL)	3536 ± 697	12041 ± 3701 *
K_el_ (h)	0.09 ± 0.02	0.06 ± 0.02
T_1/2_ (h)	8.2 ± 1.7	11.7 ± 4.3
MRT (h)	11.45 ± 2.33	16.29 ± 5.84
Relative Bioavailability (%)	100	292

*** *p* < 0.001, ** *p* < 0.005, * *p* < 0.05.

**Table 3:** Composition of developed lipid-polymer hybrid NPs (B-PLN1 to B-PLN4).

Formulae	PLGA (mg)	Tristearin (mg)	SL (mg)	BTB (mg)
B-PLN1	50	50	50	20
B-PLN2	50	100	50	20
B-PLN3	50	150	50	20
B-PLN4	50	200	50	20

## Data Availability

The data presented in this study are available on request from the corresponding author.
